# eHealth Literacy is Associated with Self-Efficacy in Adult Patients with Chronic Disease

**DOI:** 10.1093/geroni/igaf122.3215

**Published:** 2025-12-31

**Authors:** Onome Osokpo, Andrea Jackson-Sagredo, Dima Kenj Halabi, Vineet Arora, David Meltzer, Valerie Press

**Affiliations:** University of Illinois Chicago, Chicago, Illinois, United States; The University of Chicago, Chicago, Illinois, United States; The University of Chicago, Chicago, Illinois, United States; The University of Chicago, Chicago, Illinois, United States; The University of Chicago, Chicago, Illinois, United States; The University of Chicago, Chicago, Illinois, United States

## Abstract

Self-management self-efficacy—the ability to achieve positive health outcomes by successfully completing self-care tasks—is a strong predictor of effective disease self-management among patients with chronic conditions. However, little is known about how self-management self-efficacy is related to eHealth literacy (eHL), the digital skills needed to seek and use health information from electronic sources. Using a cross-sectional observational design and convenience sampling, we enrolled 1259 English-speaking hospitalized patients ≥18 years of age to test the hypothesis that self-efficacy is related to eHL. Self-efficacy was measured with a single question: “How confident are you that you can manage your health conditions on your own?” Participants responded on a five-point scale ranging from not confident at all (1) to very confident (5) (high self-efficacy: ≥4; low self-efficacy: < 3). eHL was measured with a validated eight-item questionnaire (composite score: 8–40; low eHL: < 24; adequate eHL: ≥24). The sample (54 ± 17.3 years) mostly identified as Black (73%), female (53%), single/widowed/divorced (70%), and was educated with at least some college (54%). Chronic disease was common: 29% had diabetes, 27% had heart disease, 54% had hypertension; 54% were hospitalized in the last 12 months excluding current hospitalization Unadjusted logistic regression revealed a significant association between self-efficacy and eHealth literacy (eHL) (p = 0.003). Patients with lower eHL had significantly lower odds of feeling confident in managing their medical care or illness compared to those with adequate eHL (OR = 3.62, 95% CI: 1.54–8.78, p = 0.003). Interventions promoting eHL may enhance self-efficacy, resulting in effective disease self-management.

